# Preliminary Evidence of the Efficacy of Nitric Acid Treatment in Onychomycosis

**DOI:** 10.3390/ijerph182413371

**Published:** 2021-12-19

**Authors:** Félix Marcos-Tejedor, Natividad Santos-Carnicero, Raquel Mayordomo

**Affiliations:** 1 Department of Nursing, University Center of Plasencia, University of Extremadura, 10600 Plascencia, Spain; 2 Department of Medical Sciences, Faculty of Health Sciences, University of Castilla-La Mancha, 45600 Talavera de la Reina, Spain; 3 Department of Anatomy and Human Embryology, University Center of Plasencia, University of Extremadura, 10600 Plasencia, Spain; natissa2604@hotmail.com (N.S.-C.); Rmayordo@unex.es (R.M.)

**Keywords:** onychomycosis, nails, nitric acid, treatment

## Abstract

Onychomycosis is the main cause of toenail disorders and is produced by a fungal infection. It is becoming more prevalent because of new lifestyles and immunosuppression statuses. The therapeutic approach to onychomycosis is under considerable study because of the lengthy treatments that require strong patient commitment, the limited efficacy of treatments, the inclusion of active substances that can be hepatotoxic and cause pharmacological interactions, and/or the questionable efficacy of treatments due to a lack of clinical trials. This study responds to the demand for rapid treatment with minimal pharmacological interactions. Methods: The efficacy of nitric acid 60% treatment in patients with onychomycosis was monitored and studied. The antifungal efficacy of nitric acid was measured by microbiological culture before and after treatment and the clinical evolution of nail dystrophy was quantitatively measured by monitoring with the Onychomycosis Severity Index (OSI). Results: The results show that, with the protocol used, nitric acid 60% painlessly cured 40% (microbiologic cure) of the cases treated, and in all cases, clinical improvement was observed (*p* = 0.011). Conclusions: The treatment with nitric acid 60% is as efficient as conventional treatments, requires less patient compliance of the treatment and produces no pharmacological interactions, providing alternative treatment in the case of hepatotoxicity.

## 1. Introduction

Toenail infections are among the most frequent infections in podiatry and cause clinical complications. Foot infections are normally caused by dermatophyte fungi and most commonly affect the first toe [1,2,3]. Global prevalence of onychomycosis is estimated at 2 to 8%. This range is due to factors inherent to the population studied, such as social habits, climate, age, immunosuppression status (diabetes, transplants, chemotherapy) and altered states of the circulatory and neuropathic systems [1,2,3,4]. Onychomycosis is responsible for 50% of nail disorders and can limit activities of daily life. It is also associated with a negative psychosocial impact that can cause patients to become socially isolated [5,6,7,8].

Cutaneous treatment for dermatophyte infection has a global cost of around $500 million [9]. Successful practice in the treatment of these fungal infections must take into account the severity of the infection, the infecting agent and the number of damaged nails. The correct choice of active substance is also important, depending on penetrability, keratin fixing and method of administration [10,11,12,13]. Other considerations include the efficacy/benefit cost due to possible hepatotoxicity of the antifungal treatment and the pharmacological interactions of some of the most common active substances, and follow-up and monitoring [6,12,13]. It is also necessary to assess patient hygiene, autonomy and perseverance, because antifungal pharmacological treatments are lengthy, resulting in a high rate of abandonment and failed therapy [1,13]. The systemic oral treatments with higher cure rates are terbinafine and itraconazole, and the topical treatments are Ciclopirox 8%, Efinaconazole 10% and tavaborole 5%. The need to persevere with pharmacological treatments causes patients suffering from toenail mycosis to reject treatment. This behaviour points to a social demand for treatments that require less perseverance from patients [11,12,13].

To meet this demand, recent studies have sought rapid methodologies based on attention and care from health professionals. The main areas of study are the use of laser and caustic substances originally applied in other therapies, but there is insufficient scientific evidence about these treatments in the literature [13,14,15]. Other studies have proposed new materials, such as bioceramic-fiber, or the natural extracts of the *Hypericum perforatum,* tea tree, red alga or Controlled-flux electrolyzed acidic solution to control of the skin´s microbiota, thus minimising pharmacological interaction. The authors suggest applying these solutions to population groups at high risk of infection as preventive measures for microbiological infection and the collateral damage of infection [16,17,18,19,20,21].

This study was prompted by the need for rapid and painless treatments requiring less perseverance and with minimum pharmacological interactions. We report the results obtained in an objective follow-up of onychomycosis treatment with nitric acid 60% and provide preliminary evidence based on the patients treated.

## 2. Materials and Methods

### 2.1. Patients

Five patients diagnosed with onychomycosis using traditional culture techniques—three men and two women with an average age of 53 ± 15.44 years (age range 29 to 65 years)—were treated with nitric acid 60% and monitored. Approval was obtained from the Bioethics Committee of the University of Extremadura (Ref. 07/2018). The Declaration of Helsinki was followed throughout the study and all participants signed an informed consent form.

Exclusionary criteria were established that none of the participants could have a circulatory condition or neurologic alteration that may have affected cell regeneration or prevented observation of any damage caused by the chemical. Other criteria were not being of legal age, having an active treatment for onychomycosis and having a negative microbiological culture.

### 2.2. Clinical Evaluation

The approach was clinically monitored in three ways. Firstly, a microbiological study was conducted before and after treatment to determine its efficacy, through traditional culture technique in Agar Sabouraud with chloramphenicol (Condalab, Spain) at 30 °C during three to four weeks [2,3]. To avoid environmental contamination of the samples, before taking the sample, the patients’ fingers were irrigated with 70° alcohol, and nail clippings were collected with sterile material after the complete evaporation of the disinfectant. The infecting agent was isolated by dentification of the morphological structures of the fungi performed by optical microscopy using a lactophenol blue solution as a staining agent to determine the species. Secondly, the clinical evolution of the onychomycosis was assessed before and after treatment using the Onychomycosis Severity Index (OSI) [22]. Lastly, any pain experienced during the process was assessed using the Visual Analogue Scale (VAS) [23].

### 2.3. Treatment

**Figure 1:**
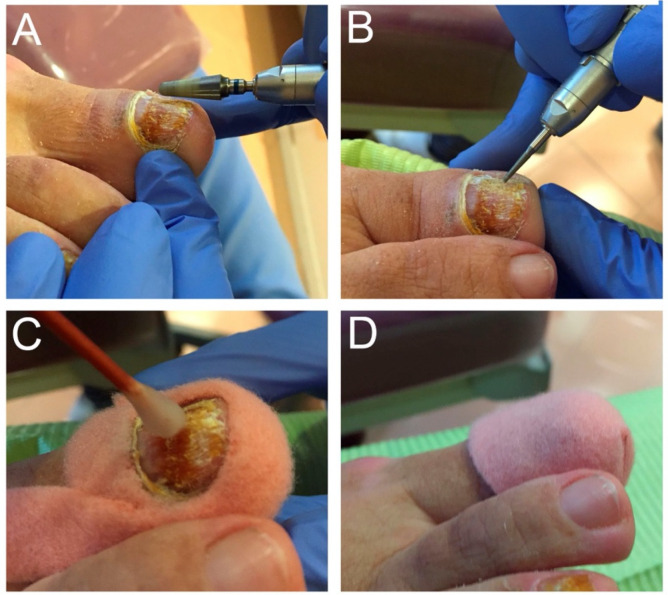
Treatment protocol: (**A**) ebridement of the affected part of the nail. (**B**) Pitting the nail plate. (**C**) Application of nitric acid 60% for 15 s. (**D**) Occlusive dressing.

Treatment comprised application of nitric acid 60 % p/p, supplied by Herbitas laboratories. Some studies used Nitric Acid for treatment of skin affected of mycosis [15]. Before treatment, as much of the affected nail as possible was debrided and small insertions were made in the remaining nail plate (Figure 1A,B). Nitric acid 60 % was applied to the entire affected area for 15 s with protection of the surrounding areas (Figure 1C), the nail was covered with an occlusive dressing and the patient was advised not to wet or change the dressing until their next visit, in 48 h (Figure 1D). Nitric acid was applied three times, with 15 days between applications. Post treatment evaluation was conducted two months after the treatment ended.

The results were analysed statistically using SPSS 15.5 (IBM, Armonk, NY, USA), and statistical significance was set at 5% (*p-value* < 0.05).

## 3. Results

All patients obtained a positive fungal culture from the nail samples collected before treatment. The culture showed that the infection was caused by a dermatophyte in three cases, yeast in one case and a mixed yeast/dermatophyte infection in one case.

**Figure 2:**
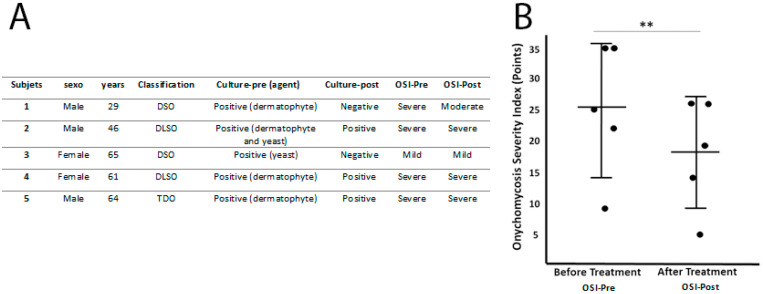
Detailed results by participants (**A**) and representation of evolution based on the points OSI (**B**). ** *p-value* = 0.011.

Based on the clinical symptoms [3], the onychomycosis was classified in two cases as distal subungual onychomycosis (DSO), in two cases as distal-lateral subungual onychomycosis (DLSO) and in one case as total dystrophic onychomycosis (TDO). According to the OSI, there were four severe cases of onychomycosis and one mild (details in the Figure 2). The mean score was 25.2 ± 10.77 points (Figure 2).

**Figure 3:**
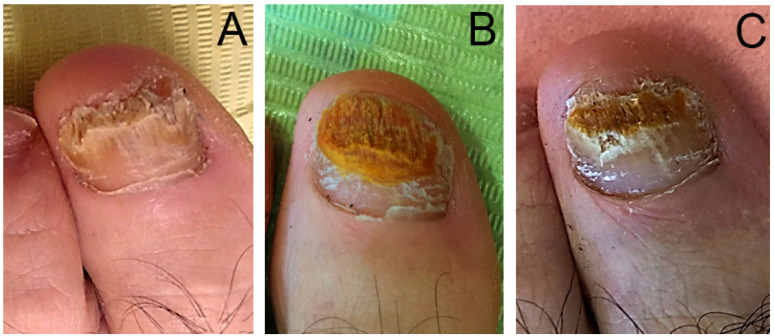
Evolution of the nail during treatment with 60% nitric acid of distal subungual onychomycosis (DSO) (**A** before–**C** after). Severe onychomycosis according to the OSI, pre-treatment. Improvement after treatment during sessions was observed (**A**–**C**) although the OSI score did vary post treatment maintains the same level of severity.

Two months after the treatment finished, we performed another nail culture and obtained 40% (n = 2) negative results and 60% (n = 3) with positive results. The negative results corresponded to the two cases of DSO caused by dermatophyte and yeast infection. During this check-up we evaluated the evolution of the clinical appearance (Figure 3) of the nail using the OSI and obtained mean scores of 18 ± 8.86 points (Figure 2), corresponding in three cases to severe onychomycosis, one to moderate and the other to mild. In none of the cases, the clinical symptoms disappeared but it was observed a clinical improvement, the three severe cases showed no change in the severity level of the OSI method, but the OSI score did vary after treatment.

Statistical analysis using the Shapiro–Wilk test showed that the sample had a normal distribution. We compared the results obtained by the OSI before and after treatment using the Student’s t-test for related samples, which showed a statistical difference with *p-value* = 0.011.

After each application of nitric acid and at the 48-h check-up, the VAS was administered and in 100% (n = 5) of cases patients said they had experienced no pain during application or post treatment.

## 4. Discussion

To enable assessment of the efficacy of onychomycosis treatment by application of nitric acid, an initial positive result was obtained in the microbiological cultures. Complementary tests are essential for therapeutic approach to onychomycosis, because clinical evidence of the nail is insufficient for accurate diagnosis and could result in unsuccessful treatment [3,5]. Despite this, microbiological study is prescribed to confirm mycosis by only 3.4% of general physicians and 39.6% of dermatologists [24].

The prevalence of these pathologies is very heterogeneous and depends on the populations studied, but it increases with age [6,24], explaining why the mean age of the population studied is around 53 years. There is high variability in the types of infecting fungi [13], influenced by the new lifestyles of contemporary society. Modern living has increased the prevalence of non-dermatophyte fungal infections and mixed infections [1,2], and this heterogeneity corresponds to the results obtained in this study in relation to the infecting agent.

After clinical confirmation of the fungal infection, another important element for successful therapeutic approach to these pathologies is objective clinical monitoring, given that clinical changes can go undetected because of the slow physiological growth of the nail plate [5,22]. The only validated method for assessing nail damage caused by onychomycosis is the OSI. We used the OSI to assess clinical damage before and after treatment, and it provided quantitative evidence (*p-value* = 0.011) of clinical improvement in all treated cases. However, the final score showed that three cases maintained the same level of severity (example in Figure 3). This is probably due to the slow growth of the nail, as previously mentioned, and because the follow-up period did not allow proper detection of displacement of the nail plate lesions towards the distal region. A longer follow-up time would enable observation of a greater reduction of the affected area and would most likely result in a different OSI score. However, we can see in Figure 3 that the most proximal area of the nail has a healthier and even broader appearance.

Negative cultures were obtained for two patients after treatment, corresponding to less severe onychomycosis. We can therefore conclude that the treatment has 40% microbiological cure. However, the clinical appearance of the nails did not worsen in any of the patients, indicating a 100% in vivo fungistatic effect. The result of this treatment is within the range of efficacy of other treatments on the market. For the pharmacological treatments available, treatment efficacy ranges from 5.5–94%, depending on the active substance and the method of administration [12,13]. For treatments with laser, very heterogeneous studies report efficacy ranging from 33–100% with microbiological treatment and 0.08–100% full recovery [14]. However, these studies provide evidence only of the clinical improvement of the treated nails [13,14]. The clinical improvement with laser and its easy administration mean that it is widely used, despite its low efficacy and The Food and Drug Administration (FDA) recommendation that this type of treatment should not be used as first-line therapy for nail fungal infection [13].

With regard to treatment of these infections with nitric acid, we found only one work that includes a series of clinical cases with 100% efficacy, although the solution applied was citric acid and sodium nitrite, for 16 weeks of treatment [15]. However, the authors did not monitor the approach as thoroughly as in the present study.

While we are aware that more studies with a higher number of samples and a longer follow-up time are needed for more robust results, in this initial work we provide significant preliminary results indicating the possibility of using a rapid, painless treatment requiring less perseverance from patients. Another aspect to take into account is that nail debridement aided therapeutic action but did not affect the post-clinical evaluation because this was only limited to treating the injured part without affecting patients’ evaluation with the OSI. The treatment has no risk of hepatotoxicity, unlike some pharmacological treatments [12], and avoids interactions with other medicines prescribed for systemic diseases with a secondary predisposition to this infection [5]. These advantages are also found with laser therapy, but laser is associated with pain during administration [14,25,26], which did not occur with the nitric acid approach shown.

## 5. Conclusions

Proper diet and the use of topical agents such as nitric acid 60% to treat onychomycosis resulted in 40% microbiological cure in the population studied, which is within the range of efficacy of other treatments. It requires limited perseverance because all patients need to visit their health professional for treatment. Moreover, it is painless and involves no risk of pharmacological interaction.

## Data Availability

Not applicable.
